# Emergence of *bla*_IMI-2_- and *bla*_IMI-16_-Producing Enterobacter asburiae in the Aquaculture Environment of Jiangsu, China

**DOI:** 10.1128/spectrum.02853-22

**Published:** 2023-03-06

**Authors:** Jie Che, Zhaoran Wang, Yuqin Song, Hongxia Guan, Min Yuan, Xia Chen, Xiaofei Zhao, Yong Xiao, Yunfei Zhang, Dan Sha, Chao Wang, Jie Feng, Juan Li

**Affiliations:** a State Key Laboratory for Infectious Diseases Prevention and Control, Collaborative Innovation Center for Diagnosis and Treatment of Infectious Disease, National Institute for Communicable Disease Control and Prevention, Chinese Center for Disease Control and Prevention, Beijing, China; b State Key Laboratory of Microbial Resources, Institute of Microbiology, Chinese Academy of Sciences, Beijing, China; c College of Life Science, University of Chinese Academy of Sciences, Beijing, China; d Wuxi Center for Disease Control and Prevention, Wuxi, China; USDA-ARS

**Keywords:** *bla*_IMI_, aquaculture environment, *Enterobacter asburiae*, novel transposon Tn*7441*

## Abstract

Carbapenem-resistant *Enterobacteriaceae* strains have emerged as a serious threat to global public health. In recent years, *bla*_IMI_, a carbapenemase gene that drew less attention before, has been increasingly detected in both clinical and environmental settings. However, the environmental distribution and transmission of *bla*_IMI_, especially in aquaculture, require systematic investigation. In this study, the *bla*_IMI_ gene was detected in fish (*n* = 1), sewage (*n* = 1), river water (*n* = 1), and aquaculture pond water samples (*n* = 17) collected from Jiangsu, China, demonstrating a relatively high sample-positive ratio of 12.4% (20/161). Thirteen *bla*_IMI-2_- or *bla*_IMI-16_-carrying Enterobacter asburiae strains were isolated from *bla*_IMI_-positive samples of aquatic products and aquaculture ponds. We also identified a novel transposon (Tn*7441*) carrying *bla*_IMI-16_ and a conserved region containing several truncated insertion sequence (IS) elements harboring *bla*_IMI-2_, all of which may play important roles in *bla*_IMI_ mobilization. The occurrence of *bla*_IMI_-carrying Enterobacter asburiae in aquaculture-related water samples and fish samples highlights the risk of transmission of *bla*_IMI_-carrying strains through the food chain and the need for effective measures to prevent further dissemination.

**IMPORTANCE** IMI carbapenemases have been detected in clinical isolates of many bacterial species with systemic infection and cause a further burden on clinical treatment in China, but their source and distribution are still unclear. The study systematically investigated the distribution and transmission of the *bla*_IMI_ gene in aquaculture-related water bodies and aquatic products in Jiangsu Province, China, which is famous for its rich water resources and developed aquaculture industry. The relatively high prevalence of *bla*_IMI_ in aquaculture samples and the identification of novel mobile elements harboring *bla*_IMI_ enhance our knowledge of *bla*_IMI_ gene distribution and highlight the public health risk and urgency of surveillance of aquaculture water systems in China.

## INTRODUCTION

Carbapenem-resistant *Enterobacteriaceae* strains have emerged as a serious threat to global public health, with a growing number of reports of these bacteria in hospitals, animals, food, and the environment (1). The “One Health” concept highlights the importance of interactions among humans, animals, and the environment to the transmission of antibiotic resistance. Among them, food production promotes the transmission of antimicrobial resistance between food-producing animals and humans (2). Accordingly, antimicrobial resistance must be surveyed and controlled in the animals used for food production and in their living environments. The abundance of antibiotic-resistant isolates or genes found in farm animals and their environment has been investigated extensively. In contrast, less attention has been paid to aquatic food products and the associated water environment, although both are important sources of farm-to-table antimicrobial resistance gene transmission.

IMIs are class A carbapenemases, with IMI-1 first described in a clinical isolate of Enterobacter cloacae in 1984 (3). Since then, IMI carbapenemases have been detected in clinical isolates of many bacterial species, such as Enterobacter asburiae, Enterobacter cloacae, Escherichia coli, Raoultella ornithinolytica, and Klebsiella variicola (4–6). Furthermore, the gene encoding IMIs, *bla*_IMI_, has been detected in environmental samples, especially those from the aquatic environment. In 2005, *bla*_IMI-2_ was identified in a self-transferable plasmid of an Enterobacter asburiae strain isolated from a river in the United States (7). That study was followed by several others in which *bla*_IMI_-producing *Enterobacteriaceae* were detected in rivers (8, 9), estuaries (10), market foods (11), and seafood (12). Thus far, 22 assigned variants of IMI enzymes have been reported (IMI-1 to -22; http://bldb.eu). However, the studies mentioned above mainly reported the emergence of strains carrying the *bla*_IMI_ gene. Only two of them demonstrated the *bla*_IMI_-positive ratios of 10% and 4% in the water ecosystems of the United States (8) and Spain (9), respectively. The epidemiological analysis for the environmental distribution and transmission of *bla*_IMI_, especially in China, has not been investigated systematically, and the mobile element(s) and specific genetic environment(s) responsible for the spread of *bla*_IMI_ remain unclear (7, 13).

In this study, we surveyed the prevalence of *bla*_IMI_ genes and their host bacteria in aquatic food products and the associated water environments in Wuxi, Jiangsu Province, China, which is famous for its aquaculture industry. A relatively high sample-positive ratio of 12.4% for *bla*_IMI_ genes was detected, and *bla*_IMI_-carrying Enterobacter asburiae strains were isolated from *bla*_IMI_-positive samples obtained from aquatic products and aquaculture ponds. A novel transposon, Tn*7441*, and a conserved region containing several insertion sequence (IS) elements were also identified, which may play important roles in the mobilization of *bla*_IMI_. Moreover, our study revealed that *bla*_IMI_-carrying Enterobacter asburiae has spread extensively in the waterbodies and aquatic products of Wuxi, highlighting the risk of *bla*_IMI_-carrying strain transmission to humans through farm-to-table processes and the need to promote effective measures to prevent further dissemination.

## RESULTS AND DISCUSSION

### Prevalence of *bla*_IMI_ in aquatic ecosystems.

Jiangsu Province is developed in the aquaculture industry. Its total fishery economy ranks second in China, and the output value of its freshwater farming ranks first. Wuxi, a city in Jiangsu Province, is surrounded by lakes of all sizes, giving rise to a large aquaculture industry. From 10 to 11 June 2019, 161 samples from aquaculture ponds, rivers, sewage, and aquatic products were collected in Wuxi and subjected to PCR analysis to detect *bla*_IMI_. Twenty (12.4%) samples were positive for *bla*_IMI_ genes, comprising 1 each from fish, sewage, and river water and 17 from aquaculture pond water (see Table S1 in the supplemental material). In mainland China, IMI-2 was reported in an Enterobacter cloacae isolate in Zhejiang in 2006 (5). Then, in 2017, IMI-3-producing Raoultella ornithinolytica RJ46C and IMI-2-producing E. coli RJ18 were identified in a hospital in Shanghai (6). Thus far, no other *bla*_IMI_-positive isolates had been reported from local healthy or clinical people. Therefore, this was a relatively high *bla*_IMI_ positivity rate reported in China, even higher than those determined in the water ecosystems of Spain (4%) (9) and the United States (10%) (8).

The *bla*_IMI_-positive samples were inoculated into culture medium to screen for *bla*_IMI_-producing bacteria, resulting in the isolation of 13 carbapenem-resistant strains from 5 samples, of which 4 were water samples collected from the fish ponds, and 1 was a fish sample obtained from another fish pond. The presence of the *bla*_IMI_ gene in the 13 strains was confirmed by PCR. Six (46%) isolates harbored *bla*_IMI-16_, and seven (54%) harbored *bla*_IMI-2_; the latter included the isolate from the fish sample. The colonies were identified using the Vitek 2 compact assay (bioMérieux, Inc.) and confirmed by 16S rRNA sequencing and whole-genome sequencing (WGS) taxonomy classification through Kraken2. All 13 strains were Enterobacter asburiae, indicating the importance of the bacterium as a carrier of *bla*_IMI_ in aquaculture. Other studies from different countries have reported *bla*_IMI_-carrying Enterobacter asburiae in clinical settings (14–16). Our results thus expand the known habitat niches of *bla*_IMI_-carrying Enterobacter asburiae.

### The *bla*_IMI_-carrying isolates were resistant to most β-lactam antibiotics.

Antimicrobial susceptibility testing showed that all 13 isolates were resistant to carbapenems (imipenem, meropenem, and ertapenem). Testing of the isolates against expanded-spectrum cephalosporins showed that all were resistant to first-generation cephalosporins (cefazolin). Some strains exhibited intermediate resistance to second-generation cephalosporins (30.8% for cefuroxime, 69.2% for cefoxitin), and all except strain CW35-4 (92.3%) were susceptible to third- and fourth-generation cephalosporins (ceftriaxone and cefepime). Additionally, in contrast to the other strains, strain CW35-4 also showed intermediate resistance to nitrofurantoin. The 13 strains exhibited full or intermediate resistance to amoxicillin-clavulanic acid and ampicillin-sulbactam. Thus, in summary, the tested strains were resistant to most β-lactam antibiotics and susceptible to several non-β-lactam antibiotics (trimethoprim-sulfamethoxazole, chloramphenicol, colistin, quinolones, aminoglycosides, and tetracyclines).

In order to further investigate the antibiotic resistance mechanism of these 13 strains, their whole-genome sequences were sequenced using Illumina HiSeq 2000. Then, the antibiotic-resistant genes (ARGs) were annotated by ResFinder. Besides *bla*_IMI_, another β-lactam antibiotic resistance gene, *bla*_ACT,_ was also detected in all the isolates. Additionally, *oqxA*/*B*, *qnrS1* (quinolone resistance), *mdf*(A) (multidrug efflux pump), and *fosA* (fosfomycin resistance) were also found to be carried by 2 to 6 isolates (Fig. S1). Furthermore, the antibiotic residues were detected for the water samples, aiming to explore whether the prevalence of antibiotic-resistant genes was related to antibiotic usage. Several commonly used antibiotics, such as chloramphenicol, sulfonamides, quinolones, macrolides, and tetracyclines, could be detected in about 2.6% to 61.5% of samples. The presence of ARGs like *oqxA*/*B*, *qnrS1*, *mdf*(A), and *fosA* in aquatic settings might be mainly under selective pressure of the detected antibiotics. However, no β-lactams were detected in any samples (Table S2). The negative detection results might be related to the rapid degradation and low dosage of lactam drugs. Nevertheless, the heavy metal residues and/or other chemicals caused by use of disinfectants in the aquaculture industry may exert coselection pressure on ARGs (17). Thus, it is difficult to suggest the selection pressure and origin of the β-lactam resistance, especially *bla*_IMI_, of the aquatic settings. Notably, it was reported that IMI had the ability to hydrolyze carbapenems but not expanded-spectrum cephalosporins. In our result, the strain CW35-4 was resistant to third- and fourth-generation cephalosporins (ceftriaxone and cefepime), which is not consistent with previous understanding and needs to be explored in future studies.

### Transfer of the *bla*_IMI-2_ gene among plasmids mediated by a conserved region.

Based on the genome sequences, multilocus sequence typing (MLST) analysis showed that the seven *bla*_IMI-2_-carrying Enterobacter asburiae strains belonged to two new sequence types (STs). The strain from the fish, CFB52, was ST1336, and the other six (85.7%) strains, isolated from the water of two fish ponds, were ST1337 (Table 1). Further pairwise homologous alignment of the *bla*_IMI-2_-carrying scaffolds of the *bla*_IMI-2_-positive strains resulted in their classification into two types, with lengths of 94 kb (strain of ST1336) and 46 kb (strain of ST1337). Strains representative of these two types of *bla*_IMI-2_-positive scaffolds, CFB52 (ST1336) and CW35-3 (ST1337), were selected for sequencing by the PacBio platform to obtain the complete genomes. According to the sequencing results, the *bla*_IMI-2_ genes were located on two plasmids, pCW353_IMI and pCFB52_IMI, carried by two Enterobacter asburiae clones. No *bla*_IMI_-carrying strain was isolated from the corresponding pond water of CFB52, which was derived from a fish sample, or, conversely, from the corresponding aquatic products from the pond waters hosting the other six isolates. Thus, whether direct transmission between the aquatic products and the aquaculture environment occurred could not be determined. Nonetheless, the high prevalence of *bla*_IMI_-carrying strains in the aquaculture environment and the contamination risk to aquatic products raise concern.

**TABLE 1:** Information and antibiotic susceptibility of *bla*_IMI_-carrying strains^*a*^

Characteristic	Data for:												
	*E. asburiae* CW19-1	*E. asburiae* CW19-2	*E. asburiae* CW19-3	*E. asburiae* CW19-4	*E. asburiae* CW35-3	*E. asburiae* CW35-4	*E. asburiae* CFB52	*E. asburiae* CW1-2.1	*E. asburiae* CW1-2.2	*E. asburiae* CW1-4.1	*E. asburiae* CW1-4.2	*E. asburiae* CW41-3.1	*E. asburiae* CW41-3.2
IMI subtype	*bla*_IMI-2_	*bla*_IMI-2_	*bla*_IMI-2_	*bla*_IMI-2_	*bla*_IMI-2_	*bla*_IMI-2_	*bla*_IMI-2_	*bla*_IMI-16_	*bla*_IMI-16_	*bla*_IMI-16_	*bla*_IMI-16_	*bla*_IMI-16_	*bla*_IMI-16_
ST	ST1337	ST1337	ST1337	ST1337	ST1337	ST1337	ST1336	ST1585	ST1585	ST1585	ST1585	ST1585	ST1585
Origin for sample	Water in fish pond 19	Water in fish pond 19	Water in fish pond 19	Water in fish pond 19	Water in fish pond 35	Water in fish pond 35	Fish in fish pond 52	Water in fish pond 1	Water in fish pond 1	Water in fish pond 1	Water in fish pond 1	Water in fish pond 41	Water in fish pond 41
MIC (μg/ml) of:													
AMC	32/16, R	32/16, R	32/16, R	32/16, R	>32/16, R	>32/16, R	>32/16, R	32/16, R	32/16, R	32/16, R	32/16, R	32/16, R	32/16, R
AMS	>16/8, I	>16/8, I	>16/8, I	>16/8, I	>16/8, I	>16/8, I	>16/8, I	>16/8, I	>16/8, I	>16/8, I	>16/8, I	>16/8, I	>16/8, I
TZP	≤4/4, S	≤4/4, S	≤4/4, S	≤4/4, S	≤4/4, S	>64/4, I	8/4, S	≤4/4, S	≤4/4, S	≤4/4, S	≤4/4, S	≤4/4, S	≤4/4, S
SXT	≤1/19, S	≤1/19, S	≤1/19, S	≤1/19, S	≤1/19, S	≤1/19, S	≤1/19, S	≤1/19, S	≤1/19, S	≤1/19, S	≤1/19, S	≤1/19, S	≤1/19, S
ATM	16, R	16, R	16, R	8, I	≤2, S	>32, R	16, R	≤2, S	≤2, S	≤2, S	≤2, S	8, I	8, I
CFZ	>16, R	>16, R	>16, R	>16, R	>16, R	>16, R	>16, R	>16, R	>16, R	>16, R	>16, R	>16, R	>16, R
CXM	16, S	8, S	8, S	8, S	8, S	>16, I	>16, I	≤4, S	≤4, S	≤4, S	≤4, S	16, I	16, I
FOX	16, S	16, S	8, S	8, S	16, I	>16, I	>16, I	>16, I	>16, I	>16, I	>16, I	>16, I	>16, I
CAZ	≤1, S	≤1, S	≤1, S	≤1, S	≤1, S	≤1, S	2, S	≤1, S	≤1, S	≤1, S	≤1, S	≤1, S	≤1, S
CRO	≤1, S	≤1, S	≤1, S	≤1, S	≤1, S	>32, R	≤1, S	≤1, S	≤1, S	≤1, S	≤1, S	≤1, S	≤1, S
FEP	≤1, S	≤1, S	≤1, S	≤1, S	≤1, S	>16, R	≤1, S	≤1, S	≤1, S	≤1, S	≤1, S	≤1, S	≤1, S
CPL	≤4, S	≤4, S	≤4, S	≤4, S	≤4, S	≤4, S	≤4, S	≤4, S	≤4, S	≤4, S	≤4, S	≤4, S	≤4, S
FOS	128, I	128, I	128, I	128, I	64, S	64, S	>128, I	≤16, S	≤16, S	≤16, S	≤16, S	≤16, S	≤16, S
IPM	>8, R	>8, R	>8, R	>8, R	>8, R	>8, R	>8, R	>8, R	>8, R	>8, R	>8, R	>8, R	>8, R
MEM	>8, R	>8, R	>8, R	>8, R	>8, R	>8, R	>8, R	8, R	8, R	4_R	4_R	8, R	8, R
ERT	>2, R	>2, R	>2, R	>2, R	>2, R	>2, R	>2, R	>2, R	>2, R	>2, R	>2, R	>2, R	>2, R
COL	≤1, S	≤1, S	≤1, S	≤1, S	≤1, S	≤1, S	≤1, S	≤1, S	≤1, S	≤1, S	≤1, S	≤1, S	≤1, S
GEN	≤2, S	≤2, S	≤2, S	≤2, S	≤2, S	≤2, S	≤2, S	≤2, S	≤2, S	≤2, S	≤2, S	≤2, S	≤2, S
TOB	≤2, S	≤2, S	≤2, S	≤2, S	≤2, S	≤2, S	≤2, S	≤2, S	≤2, S	≤2, S	≤2, S	≤2, S	≤2, S
AMK	≤8, S	≤8, S	≤8, S	≤8, S	≤8, S	≤8, S	≤8, S	≤8, S	≤8, S	≤8, S	≤8, S	≤8, S	≤8, S
NOR	≤2, S	≤2, S	≤2, S	≤2, S	≤2, S	≤2, S	≤2, S	≤2, S	≤2, S	≤2, S	≤2, S	≤2, S	≤2, S
MFX	≤0.5, S	≤0.5, S	≤0.5, S	≤0.5, S	≤0.5, S	≤0.5, S	≤0.5, S	≤0.5, S	≤0.5, S	≤0.5, S	≤0.5, S	>2, S	>2, S
CIP	≤0.5, S	≤0.5, S	≤0.5, S	≤0.5, S	≤0.5, S	≤0.5, S	≤0.5, S	≤0.5, S	≤0.5, S	≤0.5, S	≤0.5, S	1_S	1_S
LVX	≤1, S	≤1, S	≤1, S	≤1, S	≤1, S	≤1, S	≤1, S	≤1, S	≤1, S	≤1, S	≤1, S	≤1, S	≤1, S
NIT	≤16, S	≤16, S	≤16, S	≤16, S	≤16, S	>64_I	32, S	≤16, S	≤16, S	≤16, S	≤16, S	32, S	32, S
TCY	≤2, S	≤2, S	≤2, S	≤2, S	≤2, S	≤2, S	≤2, S	≤2, S	≤2, S	≤2, S	≤2, S	≤2, S	≤2, S
DOX	2, S	2, S	≤1, S	≤1, S	≤1, S	4, S	2, S	2, S	2, S	2, S	2, S	2, S	2, S
TGC	≤1, S	≤1, S	≤1, S	≤1, S	≤1, S	≤1, S	≤1, S	2, S	2, S	2, S	2, S	2, S	2, S

a AMC, amoxicillin-clavulanic acid; AMS, ampicillin-sulbactam; TZP, piperacillin-tazobactam; SXT, trimethoprim-sulfamethoxazole; ATM, aztreonam; CFZ, cefazolin; CXM, cefuroxime; FOX, cefoxitin; CAZ, ceftazidime; CRO, ceftriaxone; FEP, cefepime; CPL, chloramphenicol; FOS, fosfomycin; IPM, imipenem; MEM, meropenem; ERT, ertapenem; COL, colistin; GEN, gentamicin; TOB, tobramycin; AMK, amikacin; NOR, norfloxacin; MFX, moxifloxacin; CIP, ciprofloxacin; LVX, levofloxacin; NIT, nitrofurantoin; TCY, tetracycline; DOX, doxycycline; TGC, tigecycline; R, resistant; I, intermediate resistant; S, susceptible.

**FIG 1:**
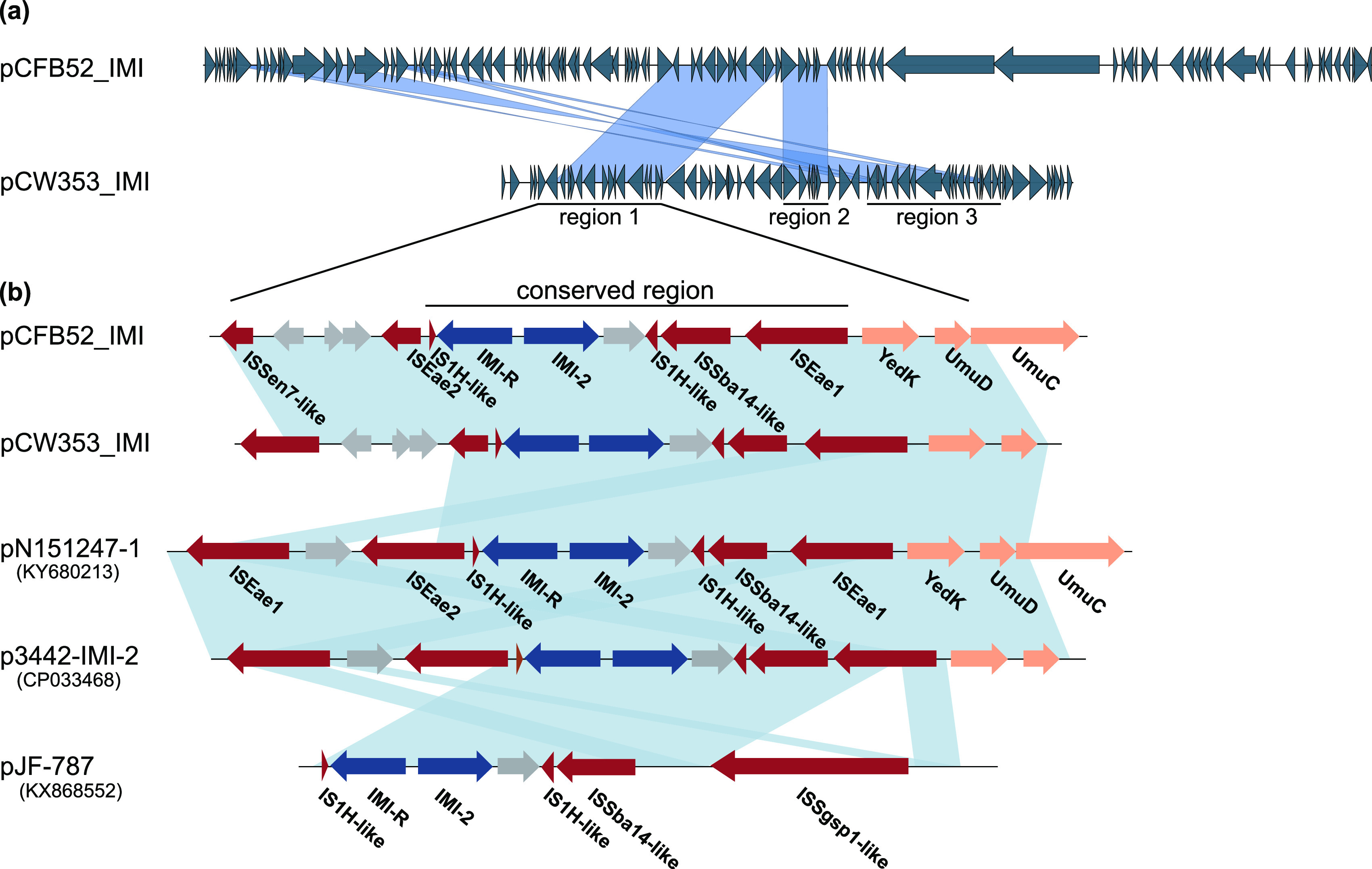
Comparative analysis of *bla*_IMI-2_-carrying plasmids. (a) Sequence comparison of two completely sequenced *bla*_IMI-2_-carrying plasmids, pCFB52_IMI and pCW353_IMI. (b) Gene environment of *bla*_IMI-2_ in different plasmids. Plasmid information is listed on the left side. Open reading frames (ORFs) are indicated by arrows. Blue arrows indicate the antimicrobial resistance gene, *bla*_IM_, and its regulator protein gene, *bla*_IMI-R_. Red arrows indicate the insertion sequences. Gray arrows indicate hypothetical proteins. Orange arrows indicate other genes. Identical regions are highlighted in light blue (>95% similarity).

pCW353_IMI, a 46,684-bp plasmid carrying *bla*_IMI-2_, has an average GC content of 51% and belongs to the IncFII (Yp) group (Fig. S2). pCFB52_IMI, a 95,423-bp plasmid carrying *bla*_IMI-2_, has an average GC content of 51% and encodes the RepA protein, which has 84.48% identity to IncFII (Yp) RepA (Fig. S3). Except for *bla*_IMI_, no other resistant gene is encoded by these two plasmids. Sequence comparisons of pCW353_IMI and pCFB52_IMI showed that they mainly shared three regions (Fig. 1a). The first (region 1) is 8,918 bp in length and contains both the *bla*_IMI-2_ and *bla*_IMI-R_ genes and many ISs flanking the two genes, including an IS*Sen7*-like element, IS*Eae2*, two IS*1H*-like elements, an IS*Sba14*-like element, and IS*Eae1*. The second region contains mainly genes encoding the UmuC subunit of DNA polymerase V, toxin, and antitoxin, while the third contains genes encoding the outer membrane usher protein FimD, chaperone protein FimC, type 1 fimbrial protein, and transposase. Alignment of pCW353_IMI and pCFB52_IMI against sequences in the NCBI nonredundant nucleotide (nr/nt) database identified three plasmids (p3442-IMI-2, pJF-787, and pN151247-1) as the best hits (identity ≥ 99%; 42% and 18% average coverage of pCW353_IMI and pCFB52_IMI with the three plasmids, respectively), as shown in Fig. 1b. Plasmid p3442-IMI-2 (GenBank accession no. CP033468) originated from Enterobacter cloacae and was isolated from Penaeus vannamei in the Netherlands (12). Plasmid pN151247-1 (GenBank accession no. KY680213) originated from Klebsiella aerogenes and was isolated in Canada from shrimp imported from Bangladesh. Plasmid pJF-787 (GenBank accession no. KX868552) originated from clinical isolates of K. variicola in the United Kingdom (4). All three plasmids were present in *Enterobacteriaceae* despite being collected from three different countries and sampled from patients and aquatic organisms, which suggests a worldwide prevalence of the *bla*_IMI-2_ gene in animals and humans. Sequence comparisons of these five *bla*_IMI-2_-carrying plasmids revealed a 5,153-bp conserved region containing *bla*_IMI-2_ and several truncated ISs (Fig. 1b). In this conserved region, the resistance gene *bla*_IMI-2_ and the LysR-type regulator gene *bla*_IMI-R_ are flanked by two truncated IS*1H*-like elements. In addition, an IS*Sba14*-like element and IS*Eae1* are found upstream of the truncated IS*1H*-like elements (Fig. 1b). Outside of this conserved region, the genetic environment of these plasmids varies widely, suggesting that pCW353_IMI and pCFB52_IMI are novel *bla*_IMI-2_-carrying plasmids. Of note, the two *bla*_IMI-2_-encoding plasmids are nonconjugative, in contrast to previously reported *bla*_IMI-2_-carrying plasmids (4, 5, 7, 12–14). The high density of IS elements flanking *bla*_IMI-2_ in the conserved region indicated that these sequences might be responsible for the mobilization of *bla*_IMI-2_ between conjugative and nonconjugative plasmids in *Enterobacteriaceae*.

### The *bla*_IMI-16_ gene is located on a novel transposon, Tn*7441*.

The *bla*_IMI-16_ genetic environment was the same among the six *bla*_IMI-16_-harboring strains of Enterobacter asburiae, and MLST analysis showed that all were ST1585. We therefore selected one strain (CW1-2.1) for sequencing by PacBio platform to obtain the complete genome. The results showed that the *bla*_IMI-16_ gene of CW1-2.1 was located on pCW1_IMI, an IncFII (Yp) group plasmid with an average GC content of 51%. pCW1_IMI contains 79 predicted open reading frames, but no resistance gene besides *bla*_IMI-16_ was found on the plasmid (Fig. S4).

**FIG 2:**
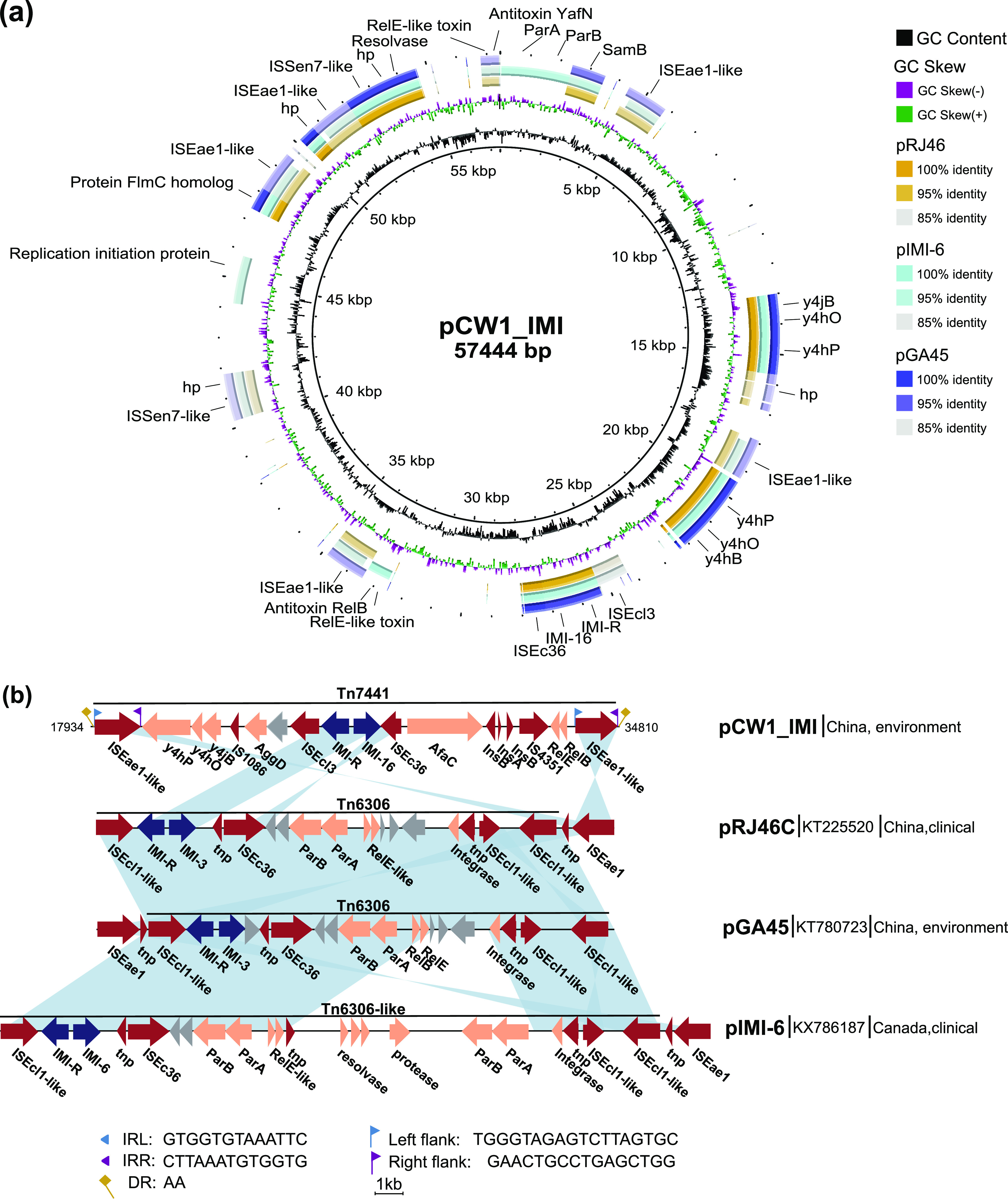
Comparative analysis of *bla*_IMI-16_-carrying plasmids. (a) BRIG comparison of pCW1_IMI with other *bla*_IMI_-carrying plasmids. The innermost rings show GC skew (purple/green) and GC content (black). Each ring represents the plasmid indicated by the corresponding color. The location of the *bla*_IMI_ gene is indicated with black text and orange shading. (b) Genetic structure of *bla*_IMI-16_ with different *bla*_IMI_-carrying plasmids. Plasmid information is listed on the right side. ORFs are indicated by arrows. Blue arrows indicate the antimicrobial resistance gene *bla*_IMI_ and its regulator protein gene *bla*_IMI-R_. Red arrows indicate the insertion sequences. Gray arrows indicate hypothetical proteins. Orange arrows indicate other genes. Identical regions are highlighted in light blue (>89% similarity).

A BLAST similarity analysis against the nr database revealed one *bla*_IMI-6_-harboring plasmid and two *bla*_IMI-3_-harboring plasmids as the best hits against pCW1_IMI, but with low coverage (42% on average and >96% identity) (Fig. 2a), suggesting that pCW1_IMI is a novel *bla*_IMI_-carrying plasmid. Plasmid pRJ46C (GenBank accession no. KT225520) was isolated from clinical strain Raoultella ornithinolytica in China, pGA45 (GenBank accession no. KT780723) was isolated from an uncultured bacterium from Chinese river sediment, and pIMI-6 (GenBank accession no. KX786187) was isolated from a clinical strain of Enterobacter cloacae in Canada. In the two *bla*_IMI-3_ plasmids (pRJ46C and pGA45), *bla*_IMI-3_ was embedded in the same Tn*6306* element, and the gene structures were similar, whereas the genetic structure of *bla*_IMI-6_ within plasmid pIMI-6 was similar to that of Tn*6306*, carrying extra genes encoding two partitioning proteins (ParA and ParB), a protease, and a resolvase (Fig. 2b). The presence of IS*Ecl1*-like elements on both ends is a feature of Tn*6306* (6). In addition, IS*Eae1* was located on one side of the Tn*6306* or Tn*6306*-like element (Fig. 2b). In our pCW1_IMI, there was only a homologous gene of the IS*Ecl1*-like element, IS*Ecl3*, located downstream of *bla*_IMI-R_-*bla*_IMI-16_. However, there were two IS*Eae1*-like genes, located ~7 kb upstream and ~6.5 kb downstream of the *bla*_IMI-16_ gene. Sequence analysis showed that these two IS*Eae1*-like genes had the same orientation, and both contained two 13-bp complete inverted repeats. Furthermore, the whole 16.8-kb region was flanked by 2-bp direct duplications immediately adjacent to each of the IS*Eae1*-like genes (Fig. 2b). This is a typical transposon structure, which is another Tn*6306*-like element. Thus, we named it Tn*7441*, according to the nomenclature of transposons (https://transposon.lstmed.ac.uk/). In Tn*7441*, several other IS elements and function genes located between the *bla*_IMI-16_ and IS*Eae1*-like genes were identified. The novel transposon Tn*7441* and other inserted sequences may play important roles in *bla*_IMI-16_ mobilization.

### Conclusions.

Compared to previous studies of the prevalence of the *bla*_IMI_ gene, the relatively high rate of *bla*_IMI_ gene detection in our samples indicates that IMI carbapenemases are dispersed in the water bodies and aquatic products of Jiangsu, China. Isolation of *bla*_IMI-2_- and *bla*_IMI-16_-producing Enterobacter asburiae strains, especially the *bla*_IMI-2_-carrying strain from fish, suggests that antibiotic resistance can be transferred from aquaculture ponds to aquatic products. A novel transposon, Tn*7441*, harboring *bla*_IMI-16_ and a conserved region in *bla*_IMI-2_-carrying plasmids were identified in this study, and they may play important roles in *bla*_IMI_ mobilization. Our study is just cross-sectional research. There are limitations in the time span and regional representation of sample collection. However, considering the risk of transferring *bla*_IMI_-carrying strains from the environment to humans through the food chain, our findings highlight the need for surveillance of resistance genes in aquatic products and in the aquaculture environment, as well as effective measures to prevent further dissemination of carbapenem resistance.

## MATERIALS AND METHODS

### Sample collection.

The sampling area is located in Ehu town of Wuxi, Jiangsu Province of China. Wuxi has Taihu Lake, the third-largest freshwater lake in China. Ehu town is near Taihu Lake, and its aquaculture industry is developed. It has a total area of about 9 square kilometers and a fishing pond area of about 2 km^2^, and it is rich in black carp, with an annual output of about 1 million kg. Meanwhile, aquatic products such as silver carp, crab, Chinese turtle, and aquatic plant Semen Euryales are all main local aquaculture species. From 10 to 11 June 2019 (during the production period), a total of 161 samples comprising 141 water, 11 fish, 7 shrimp. and 2 crabs samples were collected from aquaculture ponds (*n* = 141) in two villages (A, 31.520°N, 120.585°E; B, 31.575°N, 120.582°E) of Ehu town (see Fig. S5 in the supplemental material). About 100 mL of water samples was collected by submerging a sterile water sample collector approximately 30 cm below the water surface at three randomly selected locations in each pond. Then, the water samples from the same pond were merged into one sterile sample transport bag, resulting in a total of 141 water samples (300 mL for each). Eleven fish samples, 7 shrimp samples, and 2 crab samples were collected from 20 randomly selected ponds of the 141 ponds (one for each) and placed in sterile sample transport bags. All samples were delivered to the laboratory on ice.

We investigated the antimicrobial agent usage and water management measurements by questionnaire when sampling. Enrofloxacin, florfenicol, and sulfonamides were commonly used antibiotics during the aquatic production period, and no carbapenems were used in aquaculture and animal husbandry in China. A water purification area was set up for each fish pond. The tailwater at the end of aquaculture is first discharged into the water purification area and then discharged after ecological and physical purification by the fish, plant, and filter device in the water purification area.

### Extraction of total DNA and detection of *bla*_IMI_ genes.

For the water samples, after being fully mixed, 50 mL was taken to centrifuge at 8,000 rpm/minute for 30 min at 4°C and gentle separation of the supernatants and the sediments. The supernatants were collected in new clean tubes for antibiotic detection, while the sediments were resuspended in 1 mL sterile phosphate-buffered saline (PBS) for DNA extraction. Two-hundred-microliter sediment suspensions were used for DNA extraction by using QIAamp Fast DNA stool minikit (Qiagen, Germany). For sampled fish, crab, and shrimp, the intestines were cut open with a sterile surgical blade and rinsed in 10 mL sterile PBS. After fully mixing and discarding the tissues, the mixtures were centrifuged at 8,000 rpm/min for 30 min at 4°C, we gently discarded the supernatants, and we resuspended the sediments in 1 mL sterile PBS. We used 200 μL resuspensions for DNA extraction by using QIAamp Fast DNA stool minikit. Next, the DNA was used to detect the carbapenemase gene *bla*_IMI_ following a new high-throughput real-time PCR assay (18). In brief, gene *bla*_IMI_ detection was performed through Roche LightCycler 480 (LC 480) system. The primers and probe used in reverse transcriptase PCR (RT-PCR) are listed as follows: blaIMI-F, 5′-GGTGTCTACGCTTTAGACACTGGC-3′; blaIMI-R, 5′-CTGTGTTTAGATCT AACTCCCAACGA-3′; and blaIMI-P, FAM-TGGTCCTGAGGGTATG-MGB. The amplification conditions were 2 min at 50°C and 1 min at 95°C, followed by 40 cycles of two-step amplification of 15 s at 95°C and 1 min at 60°C.

### Determination of antibiotics in water samples.

The collected supernatants of water samples were loaded into the QTRAP 5500 liquid chromatography-tandem mass spectrometry system (AB Sciex, USA) after 0.45-μm film filtration to determine the antibiotic contents. The chromatographic condition and mass spectrum condition are outlined as follows. The determination was performed on an HSS T3 column (column length was 100 mm, inner diameter was 2.1 mm, and particle size was 1.8 μm). Phase A was 0.1% formic acid aqueous solution, and phase B was methanol. The flow rate was 0.3 mL/min. The column temperature was 40°C. The automatic injector temperature was 5°C. The injection volume was 10.0 μL. The ionization mode was positive ion electrospray ion source (ESI^+^). The ion source temperature was 550°C. The spray voltage was 5.5 kV. Curtain gas was 35 lb/in^2^. Atomizing gas was 55 lb/in^2^. Auxiliary gas was 65 lb/in^2^. The detection mode was multireactive ion monitoring mode.

### Isolation and identification of *bla*_IMI_-carrying bacteria and antimicrobial susceptibility testing.

For the *bla*_IMI_-positive water samples, the water samples were fully mixed, and a 20-mL aliquot was taken from each *bla*_IMI_-positive sample. The aliquots were centrifuged at >10,000 rpm for 2 min, and the pellet was suspended in 1 mL nutrient broth (NB). An overnight culture was prepared by inoculating 0.1 mL of the suspension into plates of *Enterobacteria* enrichment (EE) agar medium containing 0.5 mg ertapenem/L and incubating the plates at 35 ± 2°C for 18 h. As for the *bla*_IMI_-positive aquatic product samples, the intestinal content resuspensions obtained in “Extraction of total DNA and detection of *bla*_IMI_ genes” were used to isolate *bla*_IMI_-resistant strains. We plated 0.1 mL of the resuspensions on EE agar medium containing 0.5 mg/L ertapenem and incubated the plates at 35 ± 2°C overnight. All the carbapenem-nonsusceptible single colonies were selected, and colony PCR was performed to detect the *bla*_IMI_ gene. The primers and probe and the amplification conditions used in colony PCR were the same as those of RT-PCR for *bla*_IMI_ gene detection described above. Then the *bla*_IMI_-positive colonies were identified using the Vitek 2 compact assay (bioMérieux, Inc.) and confirmed by 16S rRNA sequencing and WGS taxonomy classification through Kraken2 (19). The MICs of several antibiotics against the isolated strains (Table 1) were determined using the broth microdilution method through Phoenix automated system (BD Phoenix 100, USA); with the results interpreted according to the Clinical and Laboratory Standards Institute guidelines (CLSI M100-S28) (20).

### DNA extraction, whole-genome sequencing, and plasmid analysis.

Genomic DNA of the *bla*_IMI_-carrying strains was extracted using the TIANamp bacterial DNA kit (Tiangen, China) and sequenced using Illumina HiSeq 2000. High-quality sequencing reads were *de novo* assembled using the SPAdes (v3.13.1) software (21). Strains CFB52 (ST1336), CW35-3 (ST1337), and CW1-2.1 were selected for sequencing using a single-molecule real-time (SMRT) technique on a PacBio platform (Tianjin Biochip Corporation, Tianjin, China) to obtain the complete genomes. The genomic contigs were assembled using HGAP 2.0, based on the long reads and the Illumina paired-end short reads (22). Multilocus sequence typing for Enterobacter asburiae was performed using PubMLST (https://pubmlst.org/). Plasmid replicon types were determined using PlasmidFinder (https://cge.food.dtu.dk/services/PlasmidFinder/). Aiming to predict the conjugation possibility of the *bla*_IMI_-encoding plasmids, the conjugation-related genes (including relaxase, type IV coupling protein, *virB4*/*traU* or full type IV secretion system, *oriT*, etc.) were searched through the software ICEfinder (https://bioinfo-mml.sjtu.edu.cn/ICEfinder/ICEfinder.html). Moreover, *bla*_IMI_-carrying plasmids were compared with the homologous plasmid sequences available in the NCBI database using BLAST (http://blast.ncbi.nlm.nih.gov/Blast.cgi) and visualized using BRIG (23) and Easyfig (24).

### Data availability.

The genomic sequences of the *bla*_IMI_-carrying strains used in this study are deposited in China National Microbiology Data Center (NMDC; https://nmdc.cn/en) under BioProject accession number NMDC10018005.
