# Supplementary material for: Emergence of blaIMI-2- and blaIMI-16-Producing Enterobacter asburiae in the Aquaculture Environment of Jiangsu, China
Source: Microbiol Spectr. 2023 Mar 6;11(2):e02853-22. doi: 10.1128/spectrum.02853-22 (PMC10100371; doi:10.1128/spectrum.02853-22)
Supplement: Supplemental file 3 — Figures S1-S5. Download spectrum.02853-22-s0001.pdf, PDF file, 0.6 MB [file spectrum.02853-22-s0001.pdf]

Supplementary figures:

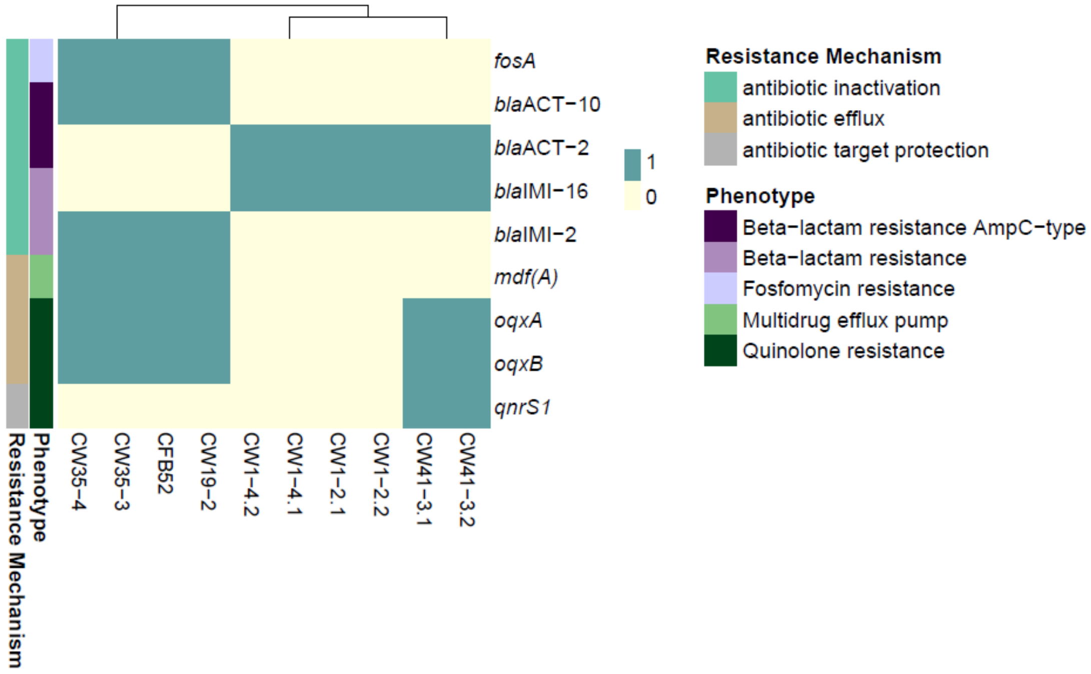

Figure S1. The heatmap for the presence (Cadet Blue) and absence (Light Yellow) of the antibiotic resistance genes (ARGs) in each strain. The resistance mechanism of each ARG and the corresponding antibiotic classes are also displayed alongside the heatmap with different colors.

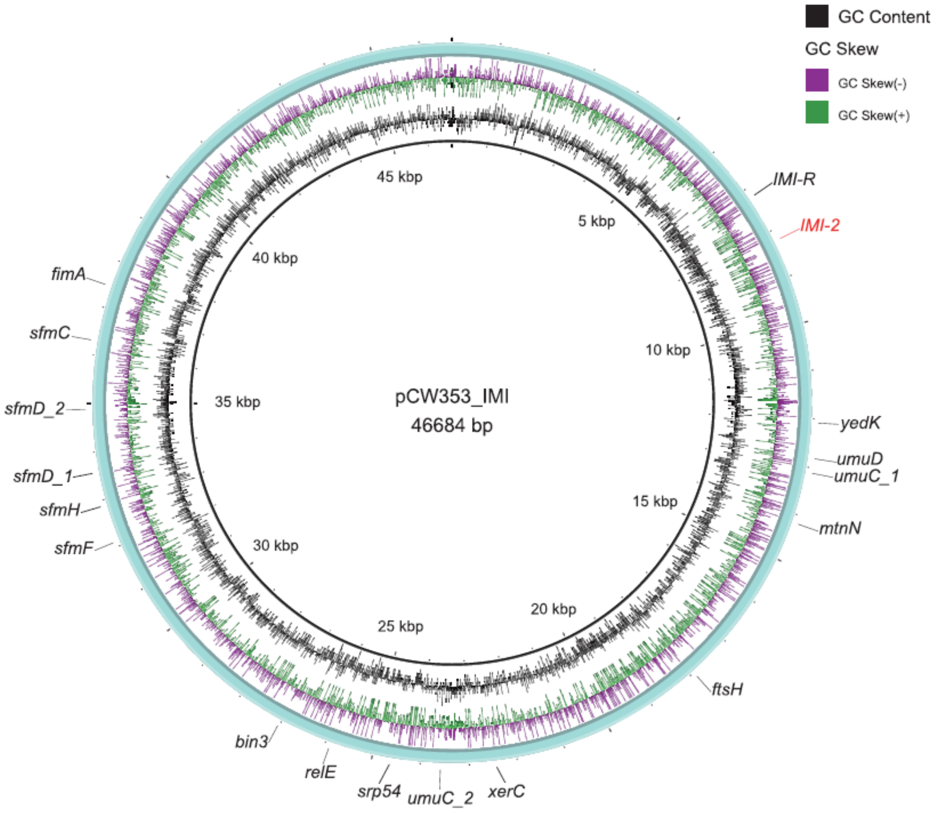

Figure S2. The circular genomic map of the plasmid pCW353_IMI. The rings from inner to outer indicated the GC content and GC skew. blaIMI and other genes annotated with known function were indicated at the corresponding location.

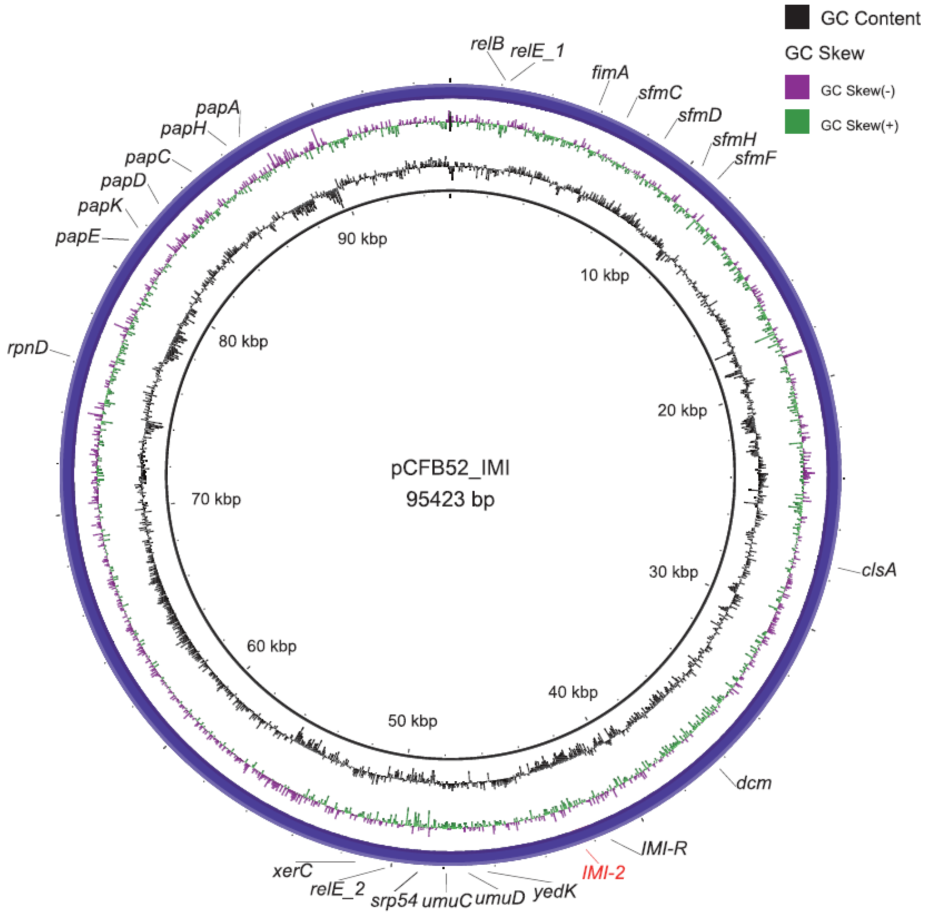

Figure S3. The circular genomic map of the plasmid pCFB52_IMI. The rings from inner to outer indicated the GC content and GC skew. blaIMI and other genes annotated with known function were indicated at the corresponding location.

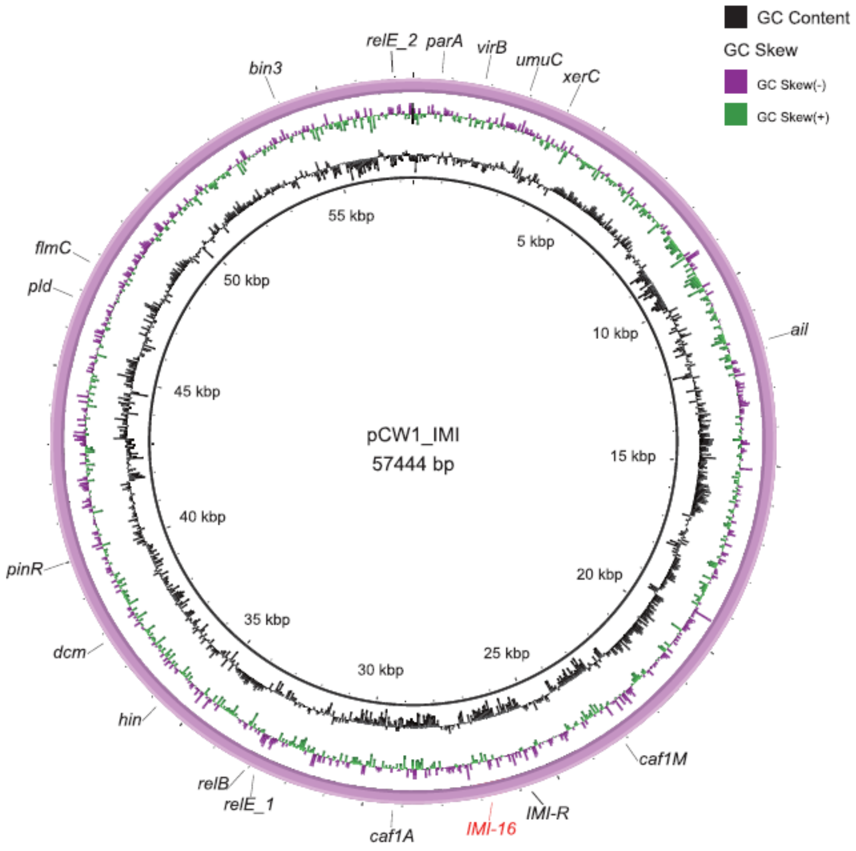

Figure S4. The circular genomic map of the plasmid pCW1_IMI. The rings from inner to outer indicated the GC content and GC skew. blaIMI and other genes annotated with known function were indicated at the corresponding location.

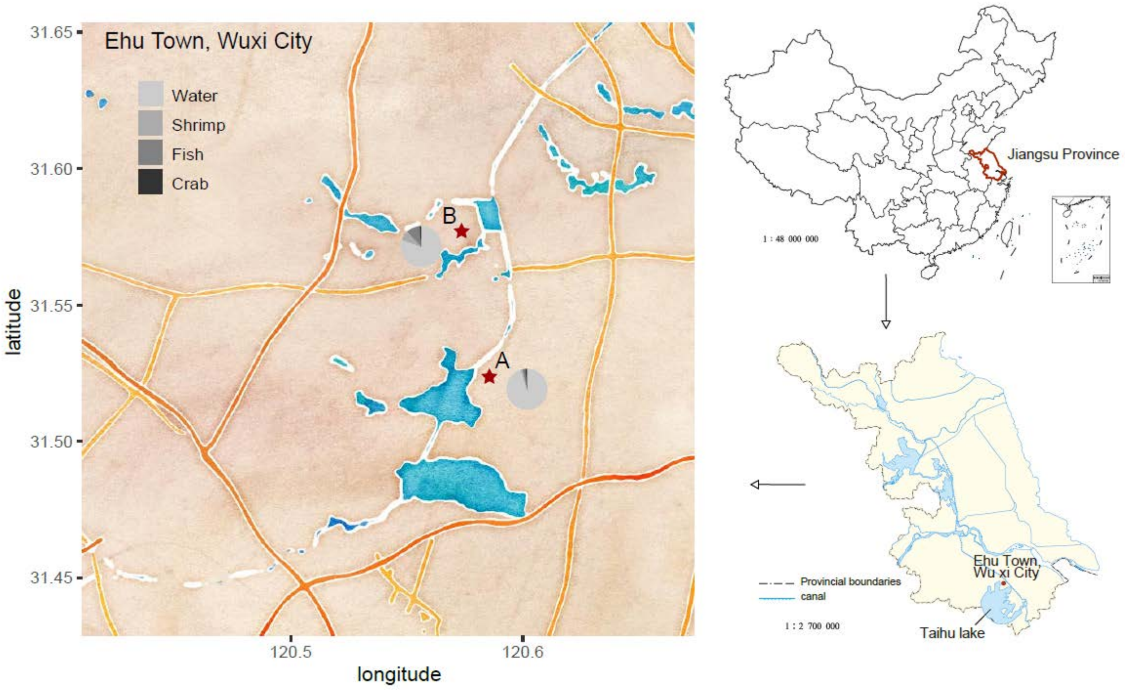

Figure S5. The locations of sampling sites. The top right corner is the map of China, and Jiangsu Province was indicated by the red outline. The lower right corner is the map of Jiangsu Province. The red circle points out the Ehu Town of Wuxi City, which is near the Taihu lake. On the left is part of the map of Ehu Town. The pentagrams represent the two main villages where we collected the samples. Blue lines and areas are rivers and lakes. Pie charts show the proportion of each sample type.
